# Identification of cuproptosis-based molecular subtypes, construction of prognostic signature and characterization of immune landscape in colon cancer

**DOI:** 10.3389/fonc.2023.927608

**Published:** 2023-03-17

**Authors:** Xu Wang, Xiaomin Zuo, Xianyu Hu, Yuyao Liu, Zhenglin Wang, Shixin Chan, Rui Sun, Qijun Han, Zhen Yu, Ming Wang, Huabing Zhang, Wei Chen

**Affiliations:** ^1^ Department of General Surgery, The First Affiliated Hospital of Anhui Medical University, Hefei, Anhui, China; ^2^ Department of Burns, The First Affiliated Hospital of Anhui Medical University, Hefei, Anhui, China; ^3^ The First Affiliated Chuzhou Hospital of Anhui Medical University, Chuzhou, Anhui, China; ^4^ Department of Biochemistry and Molecular Biology, Metabolic Disease Research Center, School of Basic Medicine, Anhui Medical University, Hefei, Anhui, China

**Keywords:** cuproptosis, colon cancer, prognosis, tumor microenvironment, immunotherapy

## Abstract

**Background:**

Cuproptosis is a newly discovered form of cell death induced by targeting lipoacylated proteins involved in the tricarboxylic acid cycle. However, the roles of cuproptosis-related genes (CRGs) in the clinical outcomes and immune landscape of colon cancer remain unknown.

**Methods:**

We performed bioinformatics analysis of the expression data of 13 CRGs identified from a previous study and clinical information of patients with colon cancer obtained from The Cancer Genome Atlas and Gene Expression Omnibus databases. Colon cancer cases were divided into two CRG clusters and prognosis-related differentially expressed genes. Patient data were separated into three corresponding distinct gene clusters, and the relationships between the risk score, patient prognosis, and immune landscape were analyzed. The identified molecular subtypes correlated with patient survival, immune cells, and immune functions. A prognostic signature based on five genes was identified, and the patients were divided into high- and low-risk groups based on the calculated risk score. A nomogram model for predicting patient survival was developed based on the risk score and other clinical features.

**Results:**

The high-risk group showed a worse prognosis, and the risk score was related to immune cell abundance, microsatellite instability, cancer stem cell index, checkpoint expression, immune escape, and response to chemotherapeutic drugs and immunotherapy. Findings related to the risk score were validated in the imvigor210 cohort of patients with metastatic urothelial cancer treated with anti-programmed cell death ligand 1.

**Conclusion:**

We demonstrated the potential of cuproptosis-based molecular subtypes and prognostic signatures for predicting patient survival and the tumor microenvironment in colon cancer. Our findings may improve the understanding of the role of cuproptosis in colon cancer and lead to the development of more effective treatment strategies.

## 1 Introduction

Cancer is the leading cause of death and reduces life expectancy worldwide (1). It is estimated that there were over 19 million new cancer cases and nearly 10 million cancer-related deaths in 2020. These cases included more than 1.9 million new cases of colorectal cancer (CRC) and 935,000 deaths, accounting for approximately one-tenth of all cancer cases and cancer-related deaths. The incidence of CRC ranks third among all cancer types, whereas its mortality rate ranks second (2). Early-stage colon cancer can be treated using surgery; however, patients with advanced colon cancer are more likely to experience metastasis and tumor recurrence, and their 5-year survival rates are less than 10% (3–5). With the development of chemotherapy and targeted medicine, the overall survival rate of patients with colon cancer has greatly improved. In recent years, advances in tumor immunotherapy and the application of immune checkpoint inhibitors (ICIs) have led to improvements in cancer treatment. Programmed cell death protein 1 (PD-1) and cytotoxic T lymphocyte antigen-4 (CTLA-4) blockade therapy are effective for treating patients with different mismatch repair metastatic CRC (6, 7).

Copper is an essential trace element in all living organisms and plays important roles in biochemical processes (8). Copper metabolism is involved in several human diseases. Wilson disease is an autosomal recessive genetic disease that mainly occurs in adolescents and is caused by a congenital disorder of copper metabolism. Patients with Wilson disease and animal models of Wilson disease show an increased incidence of hepatocellular carcinoma, suggesting that abnormal copper accumulation promotes malignant transformation through unknown mechanisms (9). Increased copper concentrations have been reported in tumors and the sera of animal models and patients, including in lung (10–12), gastrointestinal (13–18), breast (19–24), and prostate cancer (25). Resisting cell death is a basic hallmark of cancer (26). Programmed cell death is a basic physiological process that occurs in all organisms and plays a role in many biological processes, ranging from embryonic development, organ maintenance, and aging to the coordination of immune responses and autoimmunity. The effects of programmed cell death on malignant tumors, including apoptosis, autophagy, ferroptosis, pyroptosis, and necroptosis, have been widely studied. A recent study (27) reported that copper induces cell death *via* targeting lipoacylated proteins involved in the tricarboxylic acid cycle. The study showed that copper ions penetrated the mitochondria through copper carriers that directly bind to these lipoacylated proteins, causing them to form long chains and aggregate, leading to cell death. These copper ions also interfere with iron-sulfur clusters, resulting in iron-sulfur protein downregulation and leading to cytotoxic stress and death. This new form of cell death is known as cuproptosis. The authors also found that cuproptosis occured when cells were treated with the Cu ionophore elesclomol at a very low concentration, and this type of cell death can not be reversed by inhibiting necroptosis, ferroptosis, oxidative stress, and apoptosis, which indicated that cuproptosis was different from other forms of cell death. Cuproptosis may be related to various human diseases and may be a useful target for cancer therapy. It is suggested that elesclomol treatment of mice with multiple myeloma reduced the ability of cancer cells to resist the toxicity induced by proteasome inhibitors. Cu (II) bound to elesclomol interacted with mitochondrial enzyme ferredoxin 1 (FDX1) and was reduced to produce Cu (I), leading to an increase in reactive oxygen species (ROS) levels (28, 29). Xu et al. (30) reported a novel cupreous nanomaterial which could induce cuproptosis and could be used for synergistic therapy in bladder cancer. However, the effects of cuproptosis on malignant tumors remain largely unknown. Understanding how cuproptosis is initiated, propagated, and ultimately executed may be of great significance for treatment intervention and developing possible combination treatment.

With the development of high-throughput sequencing technology, researchers can access sequencing data from public databases such as The Cancer Genome Atlas (TCGA) and Gene Expression Omnibus (GEO). In recent years, many studies have focused on using sequencing data from public databases to construct tumor classifications or prognostic signatures for predicting the survival and immune landscapes of various types of malignant tumors. Chen at al (31). calculated an immune-related prognostic index for head and neck squamous carcinoma and analyzed the relationship between the index and Tumor Immune Dysfunction and Exclusion (TIDE) score and molecular subtypes. The prognostic index can be used to predict survival, immune characteristics, and the immune benefit of ICI therapy in patients with head and neck squamous carcinoma. Zhang et al. (32) classified patients with gastric cancer into three distinct molecular subtypes and constructed signatures for predicting survival and the response to immunotherapy based on the expression levels of RNA N6-methyladenosine-related genes. A previous study (33) used TCGA data to identify six immune subtypes (ISs) that encompass nearly all human malignancies, including wound healing, interferon (IFN)-γ dominance, inflammation, lymphocyte depletion, immunologically quiet, and transforming growth factor-β dominance. These six ISs are related to patient prognosis and genetic and immune characteristics, and CRC covers four of these six ISs, including wound healing, IFN-γ dominance, and inflammatory and lymphocyte depletion. Genes and long non-coding RNAs related to cell death have also been used to construct tumor classifications and prognostic signatures, including autophagy- (34), ferroptosis- (35), pyroptosis- (36), and necroptosis-related (37) genes and long non-coding RNAs. Cuproptosis is a newly discovered cell death pathway; the effects of cuproptosis-related genes on malignancies require further exploration.

In this study, we evaluated the genetic and transcriptional alterations and prognostic values of cuproptosis-related genes (CRGs) and classified patients with colon cancer into two distinct CRG clusters based on their CRG expression levels; patients were stratified into three gene clusters according to differentially expressed genes (DEGs) between two CRG clusters. A risk score was calculated to construct a prognostic signature for accurately determining the patient outcome, immune landscape, and response to immunotherapy in colon cancer. These findings improve the understanding of the role of cuproptosis in colon cancer and may enable the development of more effective treatment strategies.

## 2 Materials and methods

### 2.1 Acquisition of colon adenocarcinoma patient data

Expression profiles (fragments per kilobase million) and clinical data for were downloaded from the Genomic Data Commons Data Portal (https://portal.gdc.cancer.gov), Gene Expression Omnibus (https://www.ncbi.nlm.nih.gov/geo/, ID: GSE39582 and GSE78820), iMvigor210 (http://research-pub.gene.com/IMvigor210CoreBiologies), and Tumor Immune Dysfunction and Exclusion (TIDE) website (https://tide.dfci.harvard.edu/, ID: PRJEB25780). Fragments per kilobase million data were transformed into transcripts per kilobase million using R studio software (version 1.4.1106; The R Project for Statistical Computing, Vienna, Austria). Data from TCGA and GEO were combined, and batch effects were eliminated using *sva* package. Patients with COAD with missing clinical information were excluded from this study, and 952 patients with COAD were included after selection. The clinical characteristics of the patients are presented in **Supplementary Table S1**.

### 2.2 Genetic and transcriptional alterations of CRGs in COAD

Thirteen CRGs were recently identified (1): *FDX1*, *LIPT1*, *LIAS*, *DLD*, *DBT*, *GCSH*, *DLST*, *DLAT*, *PDHA1*, *PDHB*, *SLC31A1*, *ATP7A*, and *ATP7B* (**Supplementary Table S2**). The expression levels of CRGs in the tumor and normal tissues were compared using the Wilcoxon signed-rank test. Genomic transcriptional alterations in the 13 CRGs were analyzed. To explore CRG-related biological functions and pathways, Gene Ontology and Kyoto Encyclopedia of Genes and Genomes analyses were performed using the *ggplot2*, *Bioconductor*, and *org.Hs.eg.db* R packages.

### 2.3 Unsupervised clustering analysis of CRGs

Univariate Cox regression analysis, the Kaplan-Meier (KM) method and log-rank test were used to identify prognosis-related CRGs. Based on the 13 CRGs, consensus clustering analysis was performed using the *ConsensusClusterPlus* R package. Clustering with the highest intragroup and lowest intergroup correlations was performed to classify patients into two distinct molecular subtypes. The survival times of patients in the two identified clusters were compared. Principal component analysis was performed to distinguish between CRG clusters using the *stats* R package. The Wilcoxon test was used to compare the clinical features between the two clusters, and DEGs between CRG clusters were screened using the criteria |log fold-change| > 1 and a *p*-value < 0.05. Gene set variation analysis and single-sample gene set enrichment analysis were performed to evaluate differences in biological processes between the two clusters, immune cell infiltration, and immune-related functions using *gsva* R package.

### 2.4 Construction of cuproptosis-related prognostic risk score

Univariate Cox regression analysis was used to select prognosis-related DEGs (PRDEGs). To identify additional cuproptosis-related genes for signature construction, the patients were classified into three distinct gene clusters based on their PRDEG expression levels. The survival times, clinical characteristics, and CRG expression levels of the three gene clusters were compared, and the DEGs were identified. Least absolute shrinkage and selection operator (LASSO) regression and multivariate Cox regression analyses were performed to select CRGs for constructing the risk score using the *survival*, *survminer*, and *glmnet* R packages. The cuproptosis-related prognostic risk score was calculated based on the expression levels of the five identified CRGs. Patients with COAD were divided into high- and low-risk groups according to their risk scores. The expression of CRGs, survival status, and overall survival time of patients were compared between the high- and low-risk groups. The area under the curve (AUC) of the receiver operating characteristic (ROC) curve was used to determine the efficiency of the risk score for predicting patient survival. The risk scores between the identified clusters were compared using the Wilcoxon signed-rank test, and differences in the risk score between different risk groups based on clinical characteristics were analyzed. Univariate and multivariate Cox regression analyses were performed to determine whether the risk score was an independent prognostic factor for the prognosis of patients with COAD. A nomogram model was developed using the risk score and other clinical features, and calibration graphs were constructed to show the differences between the actual and predicted survival rates. ROC was performed to compare the prediction efficiency of the nomogram model with other clinical features.

### 2.5 Tumor microenvironment evaluation between high- and low-risk groups

To explore the relationship between the calculated risk score and TME, CIBERSORT was used to quantify the abundance of infiltrating immune cells in high- and low-risk COAD samples. Spearman’s method was used to evaluate the correlation between the risk score and immune cell abundance. The association between these immune cells and the five CRGs was also analyzed to calculate the risk score. Differences in the TME scores, including the stromal score, immune score, and ESTIMATE score, between the high- and low-risk groups were compared using Wilcoxon signed rank test.

### 2.6 Mutations, microsatellite instability, and cancer stem cell index between high- and low-risk groups

The mutation annotation format was generated using the *maftools* R package to better understand gene mutations in the two risk groups. Furthermore, the association between risk groups and the MSI and CSC index was analyzed using Wilcoxon signed rank test and the Spearman method.

### 2.7 Immune checkpoints expression, immune subtypes, and TIDE score in high- and low-risk groups

To further explore the relationship between the risk score and immune landscape in COAD, immune checkpoint expression was compared between the two risk groups. A previous study (30) described the immune landscape of various types of cancer, and COAD was classified into four distinct ISs: wound healing, IFN-γ dominant, inflammatory-depleted, and lymphocyte-depleted. The proportions of the four ISs in the high- and low-risk groups were compared using chi-square test. The TIDE scores were also compared to evaluate the potential clinical efficacy of immunotherapy in different risk groups using Wilcoxon signed-rank test.

### 2.8 Relationship between risk score and IC_50_ of therapeutic drugs

The IC_50_ is the half maximum inhibitory concentration of a drug and represents the concentration of drug required to achieve 50% inhibition of cancer cells. The IC_50_ values of nine drugs used for cancer therapy were calculated. Differences in the IC_50_ between the high- and low-risk groups were analyzed using Wilcoxon signed-rank test, and the results were shown in boxplots using *ggpubr*, *pRRophetic*, and *ggplot2* R packages.

### 2.9 Validating the risk score in immunotherapy cohorts

IMvigor210, GSE78820, and PRJEB25780 are three clinical cohorts of patients with urothelial carcinoma, melanoma, and metastatic gastric cancer who received immune checkpoints blockade therapy. Patients were divided into complete response (CR)/partial response (PR) and stable disease (SD)/progressive disease (PD) groups based on responses to immunotherapy, risk score between different groups was computed and compared using Wilcoxon signed-rank test.

### 2.10 Verifying the expression levels of five signature genes

Quantitative real-time polymerase chain reaction (qRT-PCR) was performed to verify the expression differences between normal and colon cancer tissues. Total RNA was extracted from 8 pairs of colon cancer patient tissues using TRIzol reagent (Invitrogen, Carlsbad, CA, USA). cDNA was synthesized using the total RNA and a PrimeScript RT reagent kit (Vazyme, Nanjing, China). Concentrations of cDNA samples were measured using TB Green Premix Ex Taq II (GenStar, China) with the LightCycler480 System (Applied Biosystems, Waltham, MA, United States). Relative expression levels were compulated using the 2^-ΔΔCt^ method, normalizing with GAPDH. Expression levels were compared using t-test. The primer sequences of five signature genes and GAPDH are listed in **Supplementary Table S3**. Immunohistochemistry (IHC) images were retrieved from HPA database (http://www.proteinatlas.org) to show the expression of signature genes at protein levels.

## 3 Results

### 3.1 Cuproptosis-related genes in COAD

**Figure 1:**
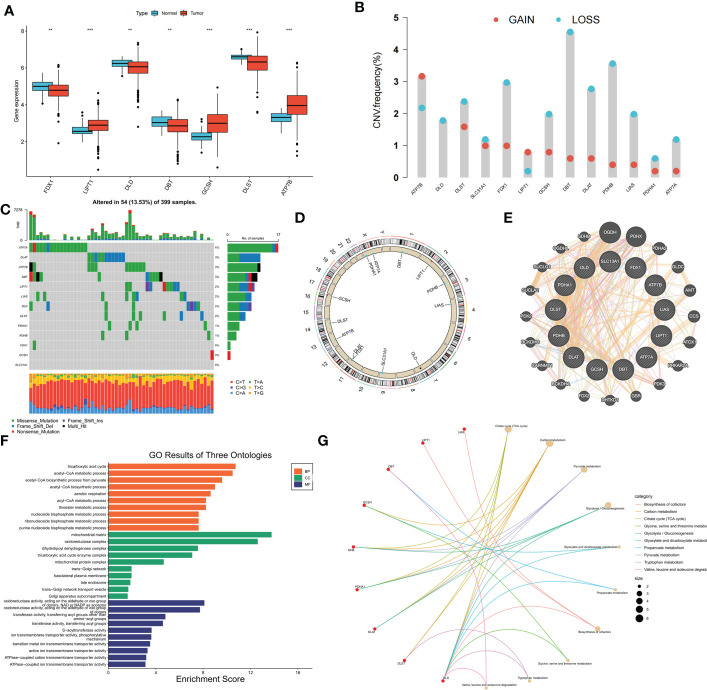
Genetic, transcriptional alterations and functional analyses of 13 cuproptosis-related genes (CRGs) in colon cancer. **(A)** Expression levels of differentially expressed CRGs between normal and tumor samples. **(B)** Mutation frequencies of 13 CRGs in colon cancer patients from TCGA cohort; **(C)** Frequencies of CNV gain, loss, and non-CNV among CRGs; **(D)** Locations of CNV alterations in CRGs on 23 chromosomes; **(E)** Protein-protein interaction network of CRGs; **(F)** GO analysis of CRGs; **(G)** KEGG analysis of CRGs. **p < 0.01; ***p < 0.001.

Gene expression data and clinical information of the patients were downloaded from the COAD project of TCGA database and GSE39582 dataset of the GEO database. Thirteen CRGs were evaluated, and the expression levels of these CRGs in the high- and low-risk groups were compared. Among the 13 CRGs, 7 were differentially expressed; *FDX1*, *DLD*, *DBT*, and *DLST* were significantly downregulated in COAD tissues, whereas *LIPT1*, *GCSH*, and *ATP7B* were upregulated in COAD tissues (**Figure 1A**). The somatic copy numbers of the 13 CRGs were analyzed, and *DBT* showed the highest copy number variation frequency (**Figure 1B**). **Figure 1C** shows the somatic mutation incidence of 13 CRGs in patients with COAD; *ATP7A* exhibited the highest mutation frequency. The locations of copy number variations in CRGs on the chromosomes are presented in **Figure 1D**. A protein-protein interaction network of the 13 CRGs was constructed using the GeneMANIA online program to determine the associations of the CRGs (**Figure 1E**). Gene Oncology (**Figure 1F**) and Kyoto Encyclopedia of Genes and Genomes (**Figure 1G**) analyses revealed significant biological processes, cellular components, molecular functions, and pathways involving the CRGs. The CRGs were mainly associated with the biological processes of the tricarboxylic acid cycle, acetyl-CoA metabolic process, and acetyl-CoA biosynthetic process from pyruvate and were correlated with the cellular components of the mitochondrial matrix, oxidoreductase complex, and dihydrolipoyl dehydrogenase complex, which are also involved in the molecular function of oxidoreductase activity, transferase activity, and *S−*acyltransferase activity. These CRGs further participate in several pathways, including biosynthesis of cofactors, carbon metabolism, and the tricarboxylic acid cycle. A network was constructed to display the interactions between the CRGs and their prognostic significance (**Supplementary Figure S1A**). Survival curves indicated that high expression of *ATP7A* is correlated with poor prognosis in patients with COAD, whereas patients with high expression of *DLAT*, *DLD*, *FDX1*, *LIAS*, *PDHA1*, *PDHB*, and *SLC31A1* had longer survival times (**Supplementary Figure S1B**).

### 3.2 Identification of CRG clusters in COAD

**Figure 2:**
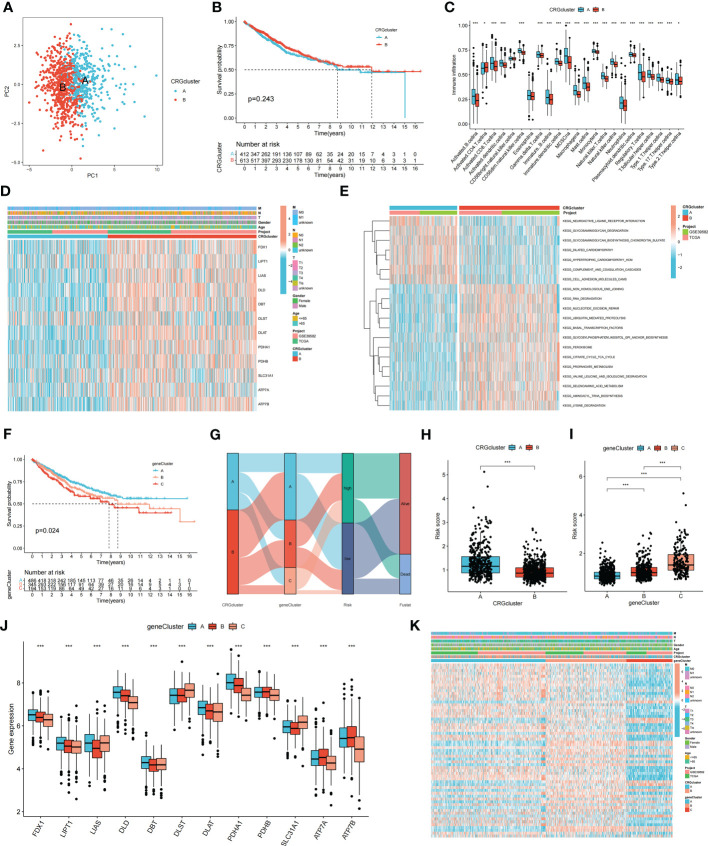
Molecular subtypes and clinical characteristics, tumor microenvironment between colon cancer samples. **(A)** PCA showed good distiction between two CRGclusters. **(B)** The KM curve revealed no significant difference in the survival time between the two clusters (*p* = 0.243). **(C)** ssGSEA investigated the differences of immune cell infiltration between two clusters. **(D)** Heatmaps showed the relationship between CRGclusters and clinical features and CRGs expression in colon cancer patients. **(E)** GSVA showed the enriched pathways in CRGclusters. *p < 0.05; ***p < 0.001. **(F)** The KM curve shows that patients in genecluster A had the longest survival time, whereas patients in genecluster C had the worst prognosis (*p* = 0.024). **(G)** Sankey plot showed the correlation between molecular classifications, risk groups and survival status in colon cancer patients. **(H, I)** Association between risk score and molecular classifications. **(J)** Expression levels of DECRGs in two geneclusters. **(K)** Heatmap showed the association between genecluster and clinical features. *p < 0.05; ***p < 0.001.

Consensus clustering analysis was performed to construct a molecular classification based on the expression levels of the CRGs. Clusters with the highest intragroup and lowest intergroup correlations were also identified. By increasing the clustering variable (k), we found that when k = 2, classification met the standard. Patients were separated into two distinct CRG clusters: A and B (**Supplementary Figure S2**). Satisfactory separation between CRG clusters A and B was observed using principal component analysis (**Figure 2A**). The KM curve revealed no significant difference in the survival time between the two clusters (**Figure 2B**). Single-sample Gene Set Enrichment Analysis was performed to evaluate differences in immune cell infiltration between CRG clusters A and B. The results suggested that CRG cluster A had higher immune cell infiltration levels, including activated B cells, activated CD4^+^ T cells, activated CD8^+^ T cells, macrophages, mast cells, and natural killer cells (**Figure 2C**). **Figure 2D** shows the correlation between CRG clusters, clinical characteristics, and CRG expression in patients with COAD. Gene Set Variation Analysis showed that CRG cluster A was significantly enriched in immune-related pathways, including neuroactive ligand receptor interaction, glycosaminoglycan degradation, glycosaminoglycan biosynthesis chondroitin sulfate, and dilated cardiomyopathy (**Figure 2E**).

### 3.3 Identification of gene clusters based on DEGs

To identify additional CRGs for calculating the risk score, gene clusters were identified. Univariate Cox regression analysis was performed to screen for PRDEGs. Patients with COAD were classified into three clusters (gene clusters A–C) according to their PRDEG expression (**Supplementary Figure S3**). **Figure 2F** shows that patients in cluster A had the longest survival time, whereas patients in cluster C had the worst prognosis (*p* = 0.024). A Sankey plot showed the relationship among CRG clusters, gene clusters, risk groups, and the living status of patients with COAD (**Figure 2G**). The risk scores in the two CRG clusters were compared; CRG cluster A had a higher risk score than CRG cluster B (**Figure 2H**). The risk score in the three gene clusters was also calculated, and the boxplot showed that gene cluster C had the highest risk score, whereas gene cluster A had the lowest risk score (**Figure 2I**). The boxplot shows that *FDX1*, *LIPT1*, *LIAS*, *DLD*, *DBT*, *DLST*, *DLAT*, *PDHA1*, *PDHB*, *SLC31A1*, *ATP7A*, and *ATP7B* were differentially expressed among the three clusters (*p* < 0.05) (**Figure 2J**). The heatmap revealed an association between gene clusters and clinical characteristics, PRDEG expression, and CRG clusters (**Figure 2K**).

### 3.4 Identification of gene clusters based on DEGs

**Figure 3:**
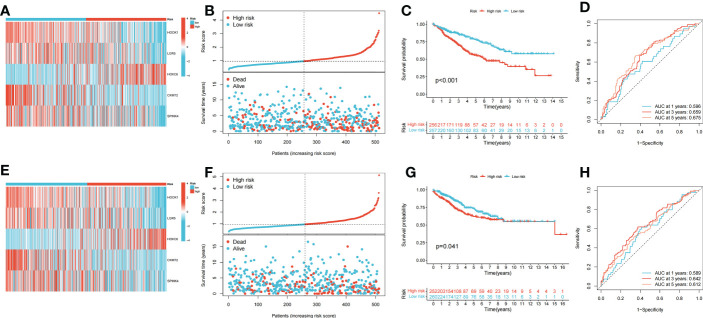
Construction and validation of the prognostic signature. **(A)** Heatmap showed the expression of 5 signature genes in two risk groups in training cohort. **(B)** Risk score and survival outcome of each case in training cohort. **(C)** KM curve showed that patients in high-risk group had a worse prognosis in training cohort (*p* < 0.001). **(D)** The AUCs for 1-, 3- and 5-year survival in training cohort. **(E)** Heatmap showed the expression of 5 genes in two risk groups in testing cohort. **(F)** Risk score and survival outcome of each case in testing cohort. **(G)** KM curve showed that patients in high-risk group had a worse prognosis in testing cohort (*p* = 0.041). **(H)** The AUCs for 1-, 3- and 5-year survival in testing cohort.

**Figure 4:**
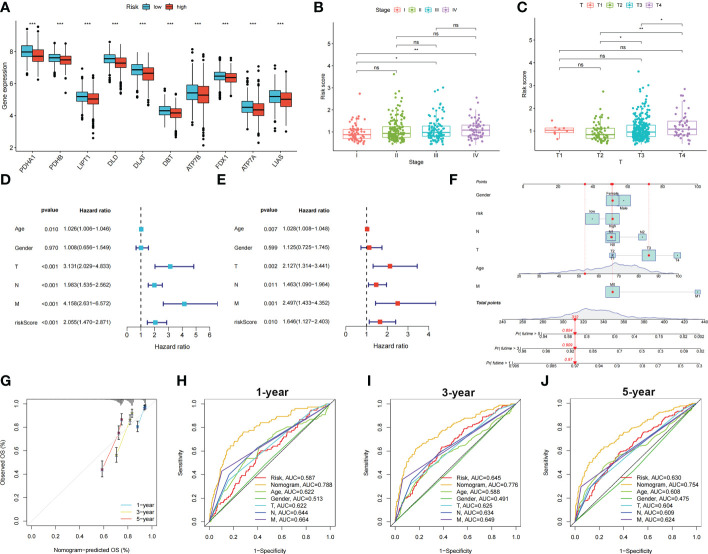
Relationship between risk score, CRGs expression and clinical features in colon cancer patients. Identification of independent prognostic factors in colon cancer and development of the nomogram model for predicting patient survival. **(A)** Expression levels of CRGs in two risk groups. Scatters diagram showed that **(B)** clinical stage and **(C)** tumor infiltration depth significantly correlated with the risk score. Forest plots of univariate **(D)** and multivariate **(E)** Cox regression analyses in colon cancer. **(F)** Nomogram using risk score and other clinical features were constructed for predicting survival of colon cancer patients. **(G)** Calibration graphs investigated that the actual survival rates of colon cancer patients were close to the nomogram-predicted survival rates. **(H–J)** 1-, 3-, and 5-year AUC showed that the nomogram had satisfactory efficiency in predicting patient survival. ns, not significant; *p < 0.05; **p < 0.01; and ***p < 0.001.

Patients with COAD were randomly divided into training and testing groups at a ratio of 1: 1. LASSO and Cox regression analyses were performed to screen CRGs to construct a prognostic signature, and five genes were included after selection. Risk scores were calculated based on the following formula: risk score = $\sum_{i=1}^{\text{n}}\beta i*\lambda i$ , where n represents the number of genes included to construct the signature and *βi* and *Λi* represent the regression coefficient and gene expression value, respectively. Patients with COAD were divided into high- and low-risk groups based on their calculated risk scores. Differences in the expression of these five genes between the two risk groups in the training group are shown in **Figure 3A**. Patients with high-risk COAD had a higher risk of mortality (**Figure 3B**). The KM plot also suggested that patients with low risk scores had a better prognosis than those with high risk scores (**Figure 3C**). ROC analysis was performed to examine the prediction efficiency of the risk score, showing AUCs for 1-, 3-, and 5-year survival of 0.596, 0.659, and 0.675, respectively (**Figure 3D**). These results were validated in the testing group (**Figures 3E–H**). *PDHA1*, *PDHB*, *LIPT1*, *DLD*, *DLAT*, *DBT*, *ATP7B*, *FDX1*, *ATP7A*, and *LIAS* showed higher expression levels in the low-risk group than in the high-risk group (*p* < 0.05) (**Figure 4A**). The risk score was correlated with tumor stage (**Figure 4B**) and infiltration depth (**Figure 4C**). Univariate (**Figure 4D**) and multivariate (**Figure 4E**) Cox regression analyses showed that the risk score is an independent prognostic factor for predicting the survival of patients with COAD (*p* < 0.05). A nomogram model was constructed based on the risk score and other clinical features (**Figure 4F**). A calibration graph was drawn to test the prediction efficiency of the nomogram model (**Figure 4G**); the predicted survival rates were similar to the actual survival rates. Prediction efficiency of the established nomogram model was compared with other clinical features, 1-, 3-, and 5-year AUC showed that the nomogram had satisfactory efficiency in predicting patient survival (**Figures 4H–J**).

### 3.5 TME evaluation between high- and low-risk groups

**Figure 5:**
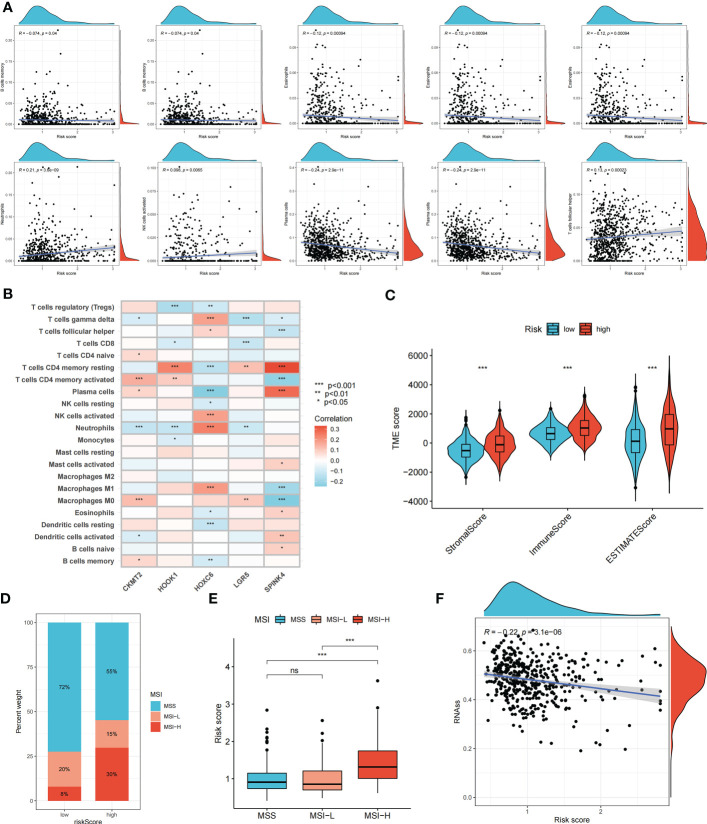
Evaluation of tumor microenvironment, MSI, and cancer stem cell (CSC) index in high- and low- risk groups. **(A)** Relationship between risk score and different immune cell types. **(B)** Correlation between the abundance of immune cells and seven genes in the prognostic signature. **(C)** Correlation between risk score and immune-related scores. **(D, E)** The relationship between the risk score and microsatellite instability (MSI) status. **(F)** The correlation between the risk score and CSC index. ns, not significant; *p < 0.05; **p < 0.01; and ***p < 0.001.

The relationship between the risk score and immune cell abundance is shown in **Figure 5A**. M0 macrophages, M1 macrophages, neutrophils, activated natural killer cells, and follicular helper T cells were positively correlated with the risk score, whereas memory B cells, resting dendritic cells, eosinophils, plasma cells, and resting memory CD4^+^ T cells were negatively correlated with the risk score. The relationship between the abundance of immune cells and the five genes in the prognostic signature is shown in **Figure 5B**. The high-risk group showed significantly higher risk scores than the low-risk group, including the stromal, immune, and ESTIMATE scores (**Figure 5C**).

### 3.6 Comparative analysis of mutations, MSI, and CSC index in high- and low-risk groups

Somatic mutations in the two risk groups of patients with COAD were compared. The five most mutated genes in the high- and low-risk groups were *APC*, *TP53*, *TTN*, *KRAS*, and *SYNE1* (**Supplementary Figure S4**). The correlation between the risk score and MSI status was analyzed (**Figures 5D, E**). A high-risk score was significantly associated with MSI-high status, whereas a low-risk score was related to microsatellite stable status. In addition, we evaluated the correlation between the risk score and CSC index values to assess the correlation between the risk score and CSCs in COAD. **Figure 5F** suggests that the risk score was negatively correlated with the CSC index (R = -0.22, *p* < 0.001).

### 3.7 Immune checkpoint expression, immune subtypes and TIDE score in high- and low-risk groups

**Figure 6:**
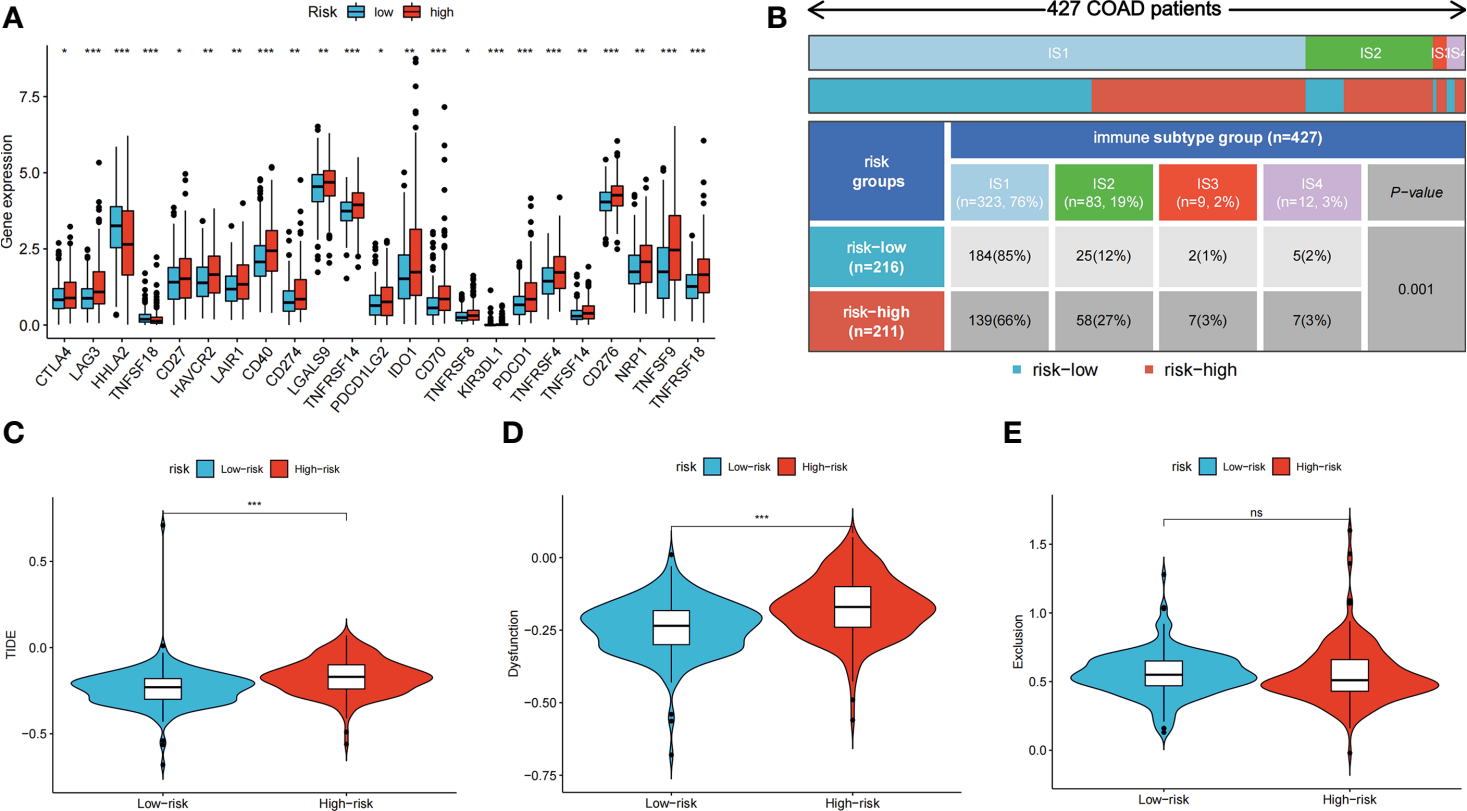
Immune checkpoint genes expression, immune subtypes distribution and TIDE score of colon cancer patients in two risk groups. **(A)** The differences of immune checkpoint gene expression in high-risk and low-risk groups. **(B)** Heatmap and table showing the distribution of colon cancer immune subtypes between two risk groups. **(C-E)** Violin plots showed the relationship between TIDE score and risk groups. ns, not significant; *p < 0.05; **p < 0.01; and ***p < 0.001.

We further explored the potential of the risk score for guiding clinical therapy for COAD. The expression levels of checkpoint genes between the high- and low-risk groups were compared. **Figure 6A** shows that immune checkpoint genes, including *CTLA-4*, *LAG3*, *CD274*, and *PDCD1*, were significantly differentially expressed between the two groups (*p* < 0.05). **Figure 6B** shows the proportion of ISs in the high- and low-risk groups; accordingly, there were more IS1 samples in the low-risk groups and more IS2, IS3, and IS4 samples in the high-risk group. The TIDE score was used to evaluate the clinical efficacy of immunotherapy in the different risk groups. A higher TIDE score indicates a higher likelihood of immune escape, suggesting that patients are less likely to benefit from ICI therapy. Our results revealed that the low-risk group had a lower TIDE score, indicating that patients in the low-risk group would show a greater benefit from ICI therapy compared to those in the high-risk group (**Figures 6C–E**).

### 3.8 Relationship between risk score and IC_50_ of therapeutic drugs

**Figure 7:**
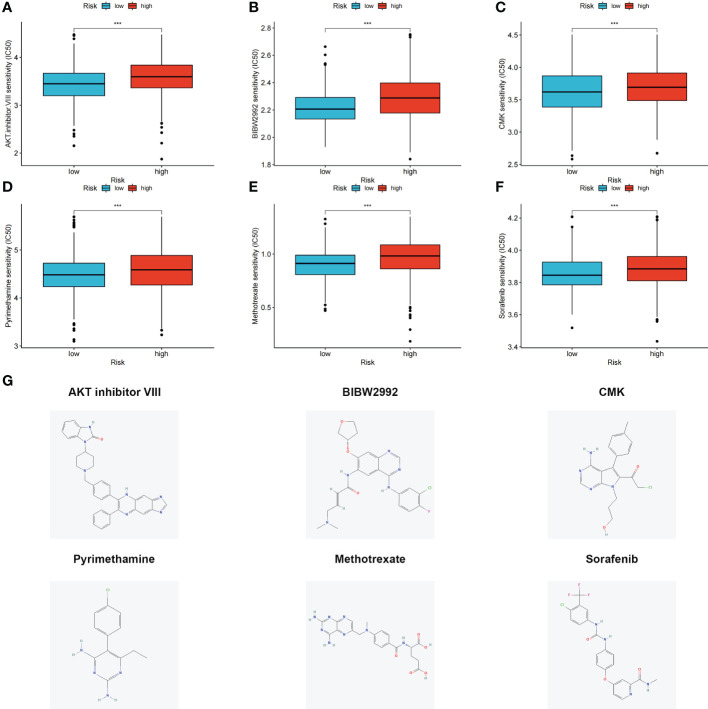
**(A–F)** Six therapeutic drugs showed significant IC50 differences in high- and low-risk groups. **(G)** 2D structures of these six therapeutic drugs. ***p < 0.001.

The differences in the IC_50_ values between the high- and low-risk groups were analyzed (**Figures 7A–F**). Lower IC_50_ values of nine drugs were associated with the risk score, and the low-risk groups showed lower IC_50_ values, suggesting that the low-risk group was more sensitive to therapeutic drugs. These results provide a reference for the clinical treatment of COAD. 2D structures of these drug molecules were also presented (**Figure 7G**).

### 3.9 Validation of the risk score in immunotherapy cohorts

**Figure 8:**
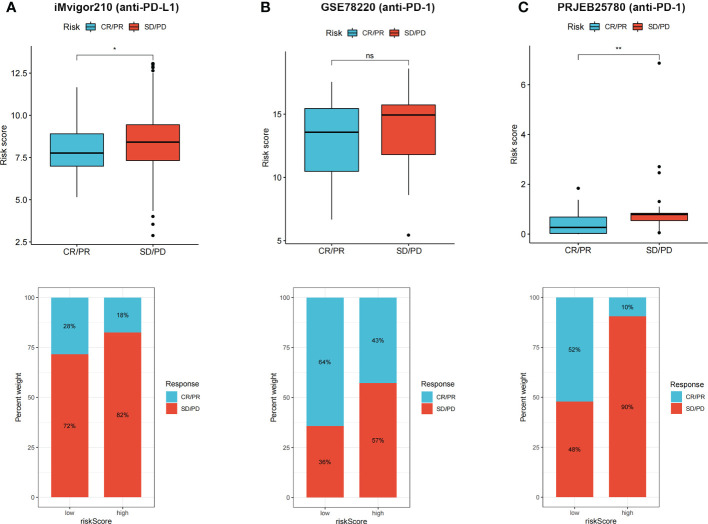
**(A–C)** CR/PR patients had lower risk score in all the three immunotherapy cohorts, and low risk group showed higher proportion of responders to anti-PD-1 and anti-PD-L1 immunotherapy. ns, not significant; *p < 0.05 and **p < 0.01.

CR/PR patients had lower risk score that SD/PD patients, and proportion of CR/PR patients was higher in low-risk group in iMvigor210 (**Figure 8A**), GSE78220 (**Figure 8B**), and PRJEB25780 (**Figure 8C**) cohorts. The results indicated that the risk score can be used to predict immunotherapy benefits.

### 3.10 Verifying the expression levels of five signature genes

**Figure 9:**
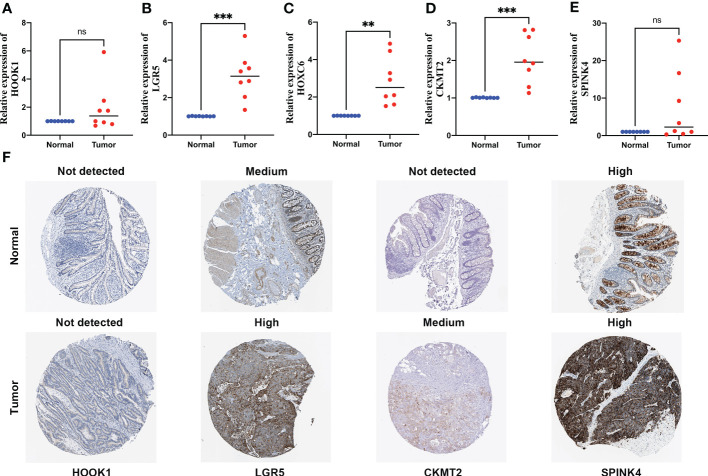
Quantitative real-time polymerase chain reaction (qRT-PCR) analyses of HOOK1 **(A)**, LGR5 **(B)**, HOXC6 **(C)**, CKMT2 **(D)** and SPINK4 **(E)** expression in 8 pairs of colon cancer tissues and adjacent non-cancer tissues, and the immunohistochemical stainings shows expression of HOOK1, LGR5, CKMT2, and SPINK4 at protein levels **(F)**. ns, not significant; **p < 0.01 and ***p < 0.001.

Among these five signature genes, HOOK1 and SPINK4 did not show significant changes in mRNA expression levels between normal and tumor tissues, whereas LGR5, HOXC6, and CKMT2 exhibited significantly increased expression in colon cancer tissues compared with that in normal tissues (**Figures 9A–E**), suggesting that these LGR5, HOXC6, and CKMT2 might be potential therapeutic targets for patients with colon cancer. IHC images of HOXC6 in colon cancer were not available in HPB database, we compared the expression differences of other four signature genes at protein levels, the expression levels were consistent with the results of qRT-PCR (**Figure 9F**).

## 4 Discussion

Trace elements are essential for human health and are involved in many biological functions, such as enzyme activity, cell signaling, and oxygen transport (38–40). Copper metabolism plays an important role in many human diseases, including in malignant tumors. A recent study (41) was performed to measure blood copper levels in 187 patients with CRC and 187 matched controls in a Polish population; the results showed that high blood copper levels were associated with an increased occurrence of CRC. Cuproptosis may be related to various types of cancer; however, its effects on colon cancer remain largely unknown.

Thirteen CRGs were identified in a previous study (27). Some CRGs were correlated with malignant tumors. *FDX1* was reported to be related to the prognosis of patients with lung cancer (42). The antisense regulation of *GCSH* can determine the viability of breast cancer cells (43). Increased expression of *ATP7A* correlates with platinum resistance in esophageal squamous cell cancer (44). *APT7A* and *ATP7B* have also been reported as predictive markers of platinum resistance in ovarian cancer (45). Genetic and transcriptional alterations of 13 CRGs in colon cancer were explored, and we determined the relevant biological functions and pathways of these CRGs. The prognostic values of CRGs were also analyzed, and eight CRGs were correlated with the survival of patients with colon cancer.

Expression and clinical data were used to classify patients with colon cancer into two distinct CRG clusters based on their CRG expression levels. CRG cluster A showed higher immune cell infiltration levels. Tumor-infiltrating immune cells can affect the response to anti-checkpoint blockade. A previous study (46) reported that tumor-infiltrating CD4^+^ T cells can upregulate some immune checkpoint genes, including PD-1, T-cell immunoglobulin, mucin domain-3, cytotoxic T lymphocyte associated protein-4, and lymphocyte-activation-gene-3. PRDEGs between CRG clusters A and B were identified, and patients were divided into three distinct gene clusters according to the expression values of the PRDEGs. Patients with gene cluster A had the longest survival time, whereas those in cluster C had the worst outcomes.

Multivariate Cox and LASSO regression analyses were performed to screen for CRGs to construct a prognostic risk signature. The risk score was calculated based on the expression levels of five genes: *HOOK1*, *LGR5*, *HOXC6*, *CKMT2*, and *SPINK4*. *HOOK1* expression is related to histologic variants, the maximum tumor diameter, and intrathyroidal dissemination in patients with thyroid carcinoma (47). *LGR5* has been identified as a strong CSC biomarker in CRC (48). Overexpression of *HOXC6* is significantly associated with high immunogenicity in non-metastatic CRC (49). High *SPINK4* expression is associated with advanced clinicopathological features and a poor response to neoadjuvant concurrent chemoradiotherapy in patients with rectal cancer (50).

Based on the calculated risk score, the patients were divided into high- and low-risk groups. Low-risk patients had a significantly longer survival time compared to high-risk patients. The relationship between the risk score and two CRG clusters, three gene clusters, CRGs expression, and clinical features was analyzed. CRG cluster A had higher risk score compared to the CRG cluster B; gene cluster C showed the highest risk score, whereas gene cluster A had the lowest risk score. Ten CRGs showed higher expression levels in the low-risk group. The risk score was correlated with tumor stage and tumor infiltration depth, indicating that the risk score can be used to predict the occurrence and development of colon cancer. Analysis of the role of clinical variables and the risk score for predicting the prognosis of patients with colon cancer patients showed that the risk score remained significant after these analyses, suggesting that the calculated risk score was an independent prognostic factor for predicting patient survival. Nomograms are widely used as tools in oncology, particularly for survival prediction (51, 52). A nomogram model was developed based on the risk score and other clinical characteristics, and calibration graphs showed that the predicted survival rates were similar to the actual survival rates, indicating that the nomogram model has a high prediction efficiency.

The TME consists of cellular components, including fibroblasts, endothelial cells, and immune cells, such as macrophages, myeloid-derived suppressor cells, and lymphocytes, and non-cellular components, including matrix proteins, cytokines, growth factors, nucleic acids, and metabolites (53). A previous study (54) suggested that the TME plays an important role in tumor development, progression, and resistance to therapeutic drugs. The correlation between the risk score and immune cells was analyzed, and five types of immune cells were positively related to the Wisk score, whereas the other five types of immune cells were negatively correlated with the risk score. *CKMT2*, *HOOK1*, *HOXC6*, *LGR5*, and *SPINK4* were significantly associated with various types of immune cells. An immune score based on immunogenomic analysis can indicate the efficacy of immunotherapy and chemotherapy (55). TME scores, including stromal, immune, and ESTIMATE scores, showed significant differences in high- and low-risk groups, suggesting that the risk score can be used to predict the response to immunotherapy and chemotherapy in patients with colon cancer.

MSI is caused by different mismatch repair mechanisms, which are strongly associated with the response to PD-1 blockade therapy (56). Patients with MSI-high/different mismatch repair CRC do not greatly benefit from neoadjuvant chemoradiotherapy or neoadjuvant chemotherapy (57). A high-risk score was found be significantly associated with an MSI-high status in patients with colon cancer, suggesting that high-risk patients would benefit less from neoadjuvant chemoradiotherapy or neoadjuvant chemotherapy. CSCs are a subset of tumor cells associated with tumor metastasis, recurrence, and drug resistance. CSCs exhibit self-renewal and differentiation abilities similar to those of normal stem cells (58). The risk score was related to the CSC index, indicating that the risk score is related to colon cancer progression. Differences in immune checkpoint gene expression between the high- and low-risk groups were also analyzed. The expression levels of the checkpoints significantly differed between the two groups. The correlations between risk groups and previously identified immune subtypes of colon cancer indicated that there was more wound healing and fewer lymphocyte depletion, inflammatory, and IFN-γ-dominant samples in the high-risk group compared to in the low-risk group. The high-risk group showed a higher TIDE score, indicating a higher likelihood of immune escape, and high-risk patients were less likely to benefit from ICI therapy. The low-risk group had lower IC_50_ values for nine types of therapeutic drugs, suggesting that low-risk patients may be more sensitive to immunotherapeutic and chemotherapeutic drugs. To validate our findings in an external cohort, the relationship between the risk score and patient survival and response to immunotherapy was explored using immunotherapy cohorts. Patients with complete/partial responses had lower risk scores, indicating that low-risk patients would achieve better immunotherapeutic effects in response to immunotherapy compared to those of high-risk patients. These results validated the efficiency of the risk score for predicting patient outcomes and responses to immunotherapy.

However, our study had some limitations. First, our analysis was based on public datasets and retrospectively collected samples, which may have caused inherent case selection bias. Second, further *in vitro* and *in vivo* experiments are required to validate our findings. Finally, clinical features related to surgery, neoadjuvant chemotherapy, and tumor markers were not considered. Thus, more clinical cases must be evaluated to confirm our conclusions.

The cuproptosis-based molecular subtypes and prognostic signature may be useful for predicting survival, TME, and guiding clinical therapy for colon cancer. Our findings may improve the understanding of cuproptosis in colon cancer and suggest more effective treatment strategies. However, additional experiments should be performed and clinical cases must be evaluated to validate our findings and further explore the effects of cuproptosis on colon cancer.

## Data availability statement

The datasets presented in this study can be found in online repositories. The names of the repository/repositories and accession number(s) can be found in the article/**Supplementary Material**.

## Ethics statement

The studies involving human participants were reviewed and approved by The Ethics Committee of The First Affiliated Hospital of Anhui Medical University. The patients/participants provided their written informed consent to participate in this study.

## Author contributions

XW, XZ and XH are responsible for writing and submitting the manuscript. YL, ZW, SC, and RS are responsible for data collection and analysis. QH, ZY and MW are responsible for the production of pictures. HZ and WC are responsible for the ideas and guidance. All authors contributed to the article and approved the submitted version.

## Supplementary material

Supplementary Table 1 Clinical information of 952 colon cancer patients from TCGA and GEO database.

Supplementary Table 2 List of 13 cuproptosis-related genes.

Supplementary Table 3 Primer sequences for PCR amplification.

Supplementary Table 4 Data and scripts used in the study were uploaded to jianguoyun, the link is included.
