# Supplementary figures and images for: Identification of cuproptosis-based molecular subtypes, construction of prognostic signature and characterization of immune landscape in colon cancer
Source: Front Oncol. 2023 Mar 17;13:927608. doi: 10.3389/fonc.2023.927608 (PMC10064275; doi:10.3389/fonc.2023.927608)

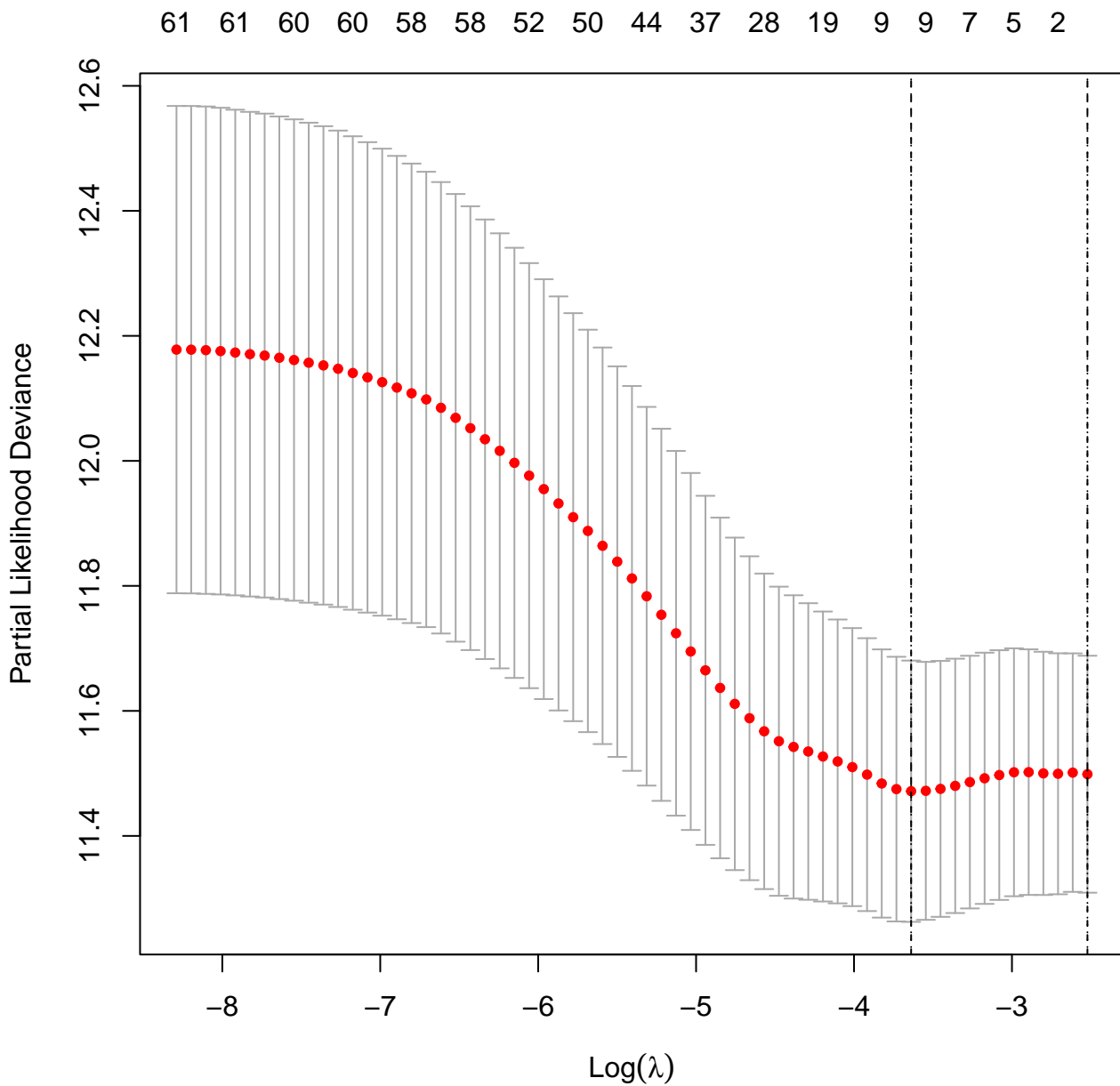

Supplement: Supplementary file 5 [file DataSheet_2.zip › Data Sheet 2/lasso.cvfit.pdf]

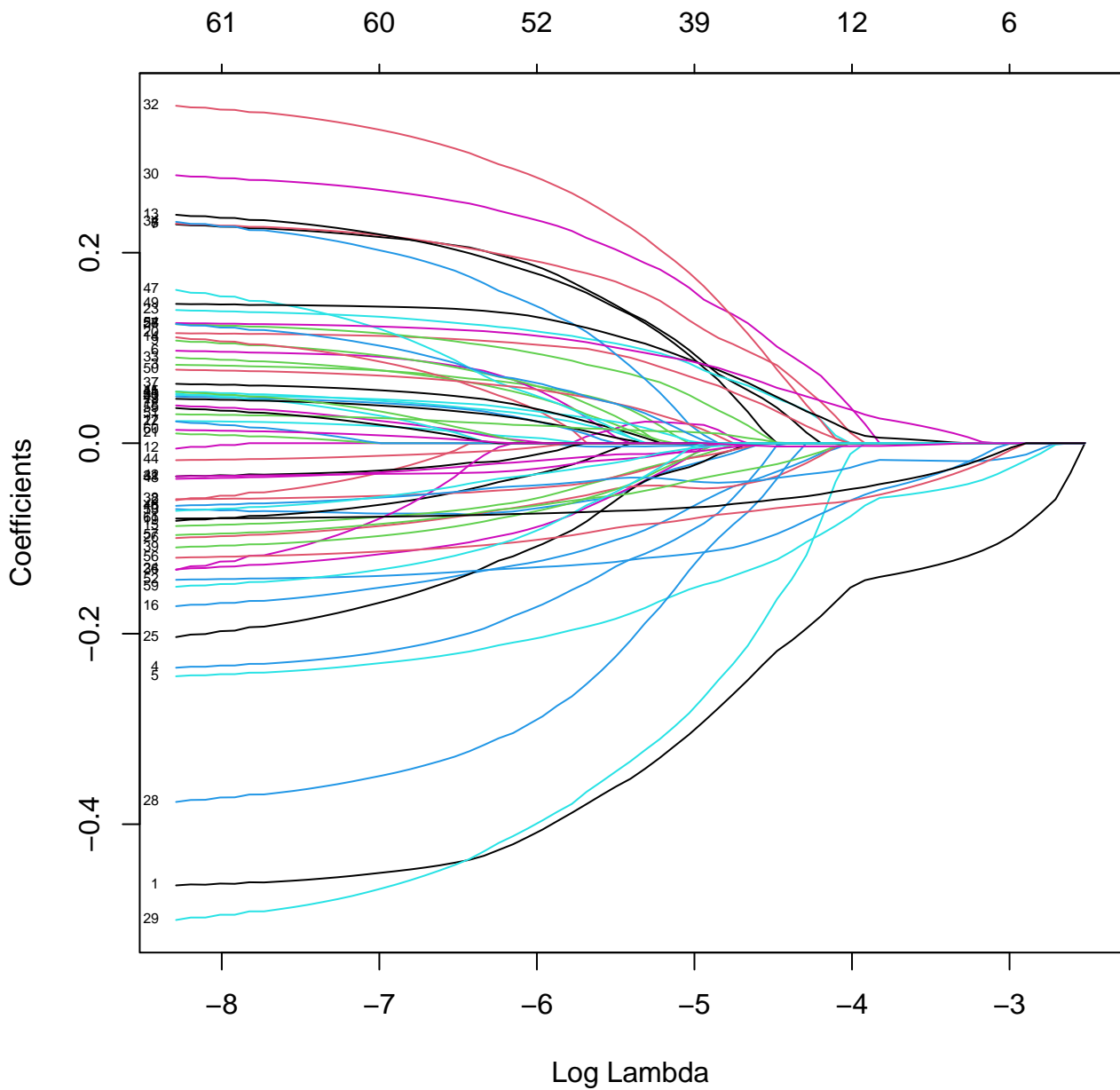

Supplement: Supplementary file 5 [file DataSheet_2.zip › Data Sheet 2/lasso.lambda.pdf]
