# Supplementary material for: Identification of cuproptosis-based molecular subtypes, construction of prognostic signature and characterization of immune landscape in colon cancer
Source: Front Oncol. 2023 Mar 17;13:927608. doi: 10.3389/fonc.2023.927608 (PMC10064275; doi:10.3389/fonc.2023.927608)
Supplement: Supplementary file 6 [file Image_1.pdf]

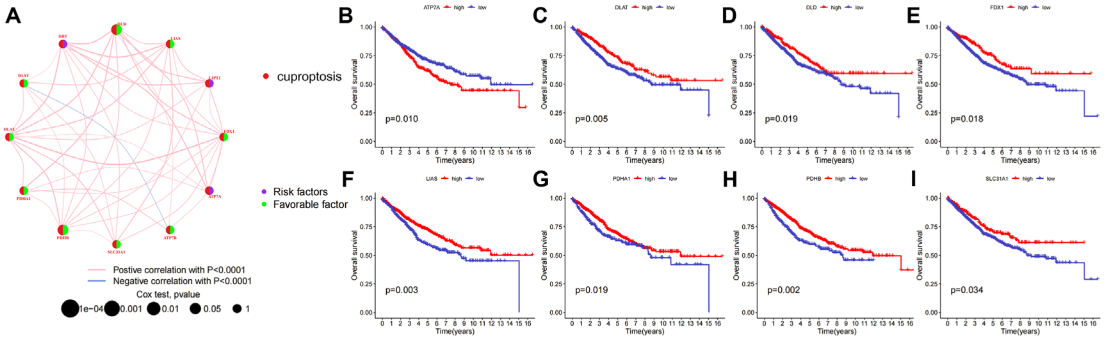

Supplementary Figure S1: Prognostic significance of CRGs. (A) Interactions among CRGs in colon cancer. The lines among the CRGs represents their interactions. Blue and red represent negative and positive correlations. (B-I) Kaplan–Meier curves of the relationship between CRGs expression and the prognosis of colon cancer patients based on TCGA and GEO databases ($p < 0.05$).

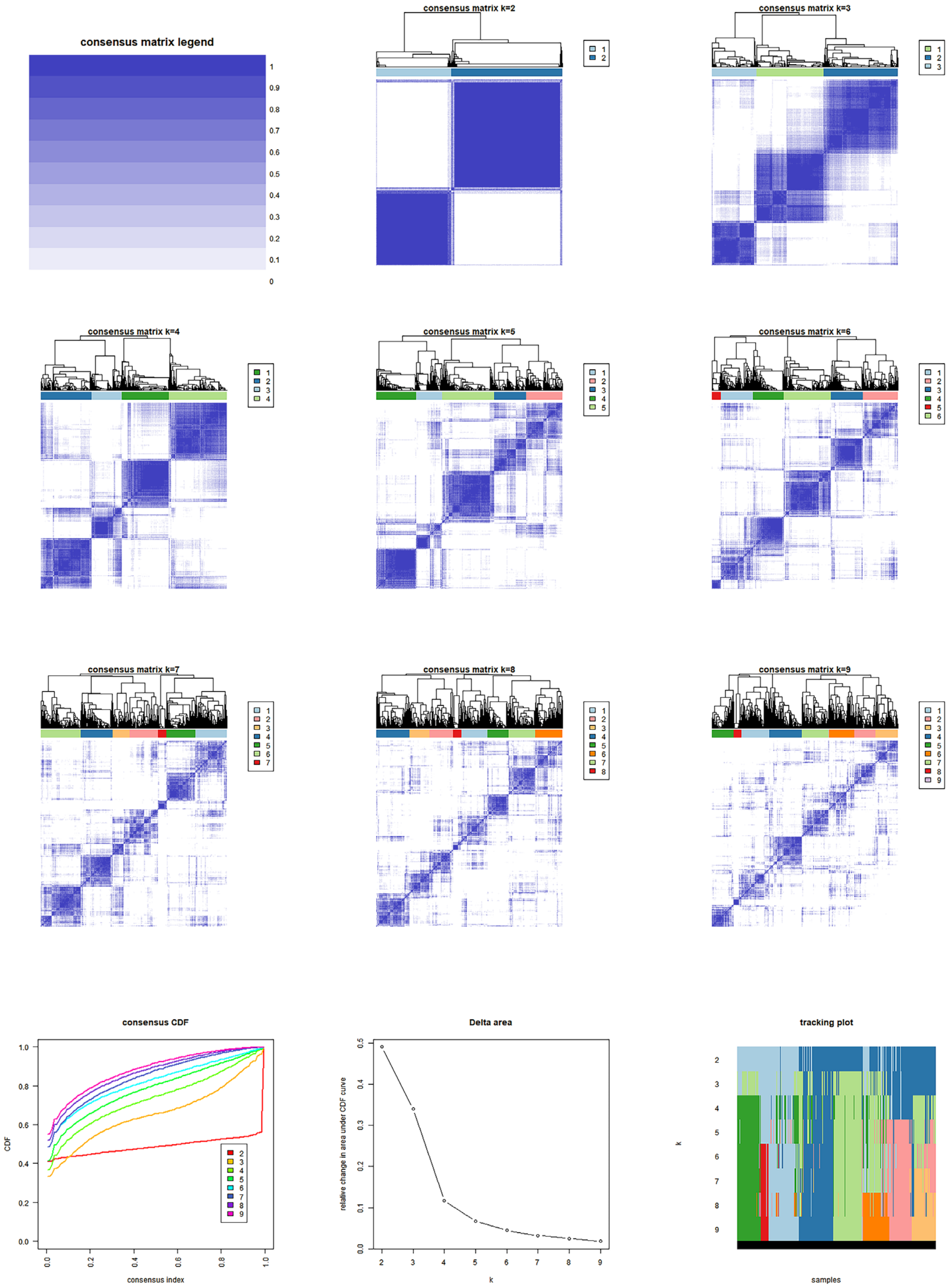

Supplementary Figure S2: Unsupervised clustering of cuproptosis-related genes and Consensus matrix heatmaps for k = 1-9. Two CRGclusters were defined using consensus clustering analyses.

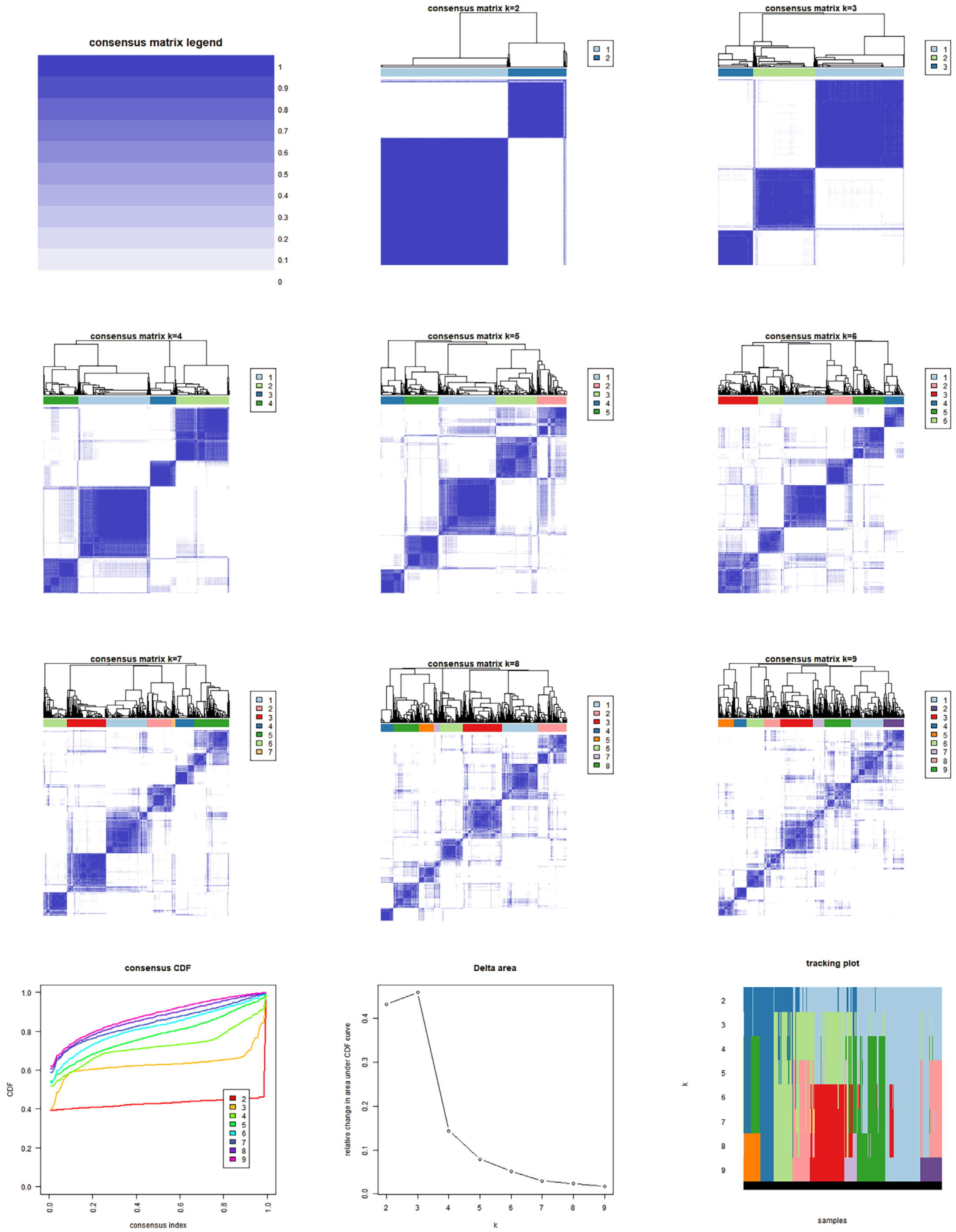

Supplementary Figure S3: Gene clustering and Consensus matrix heatmaps for k = 1-9. Three geneclusters were identified based on differentially expressed genes (DEGs).

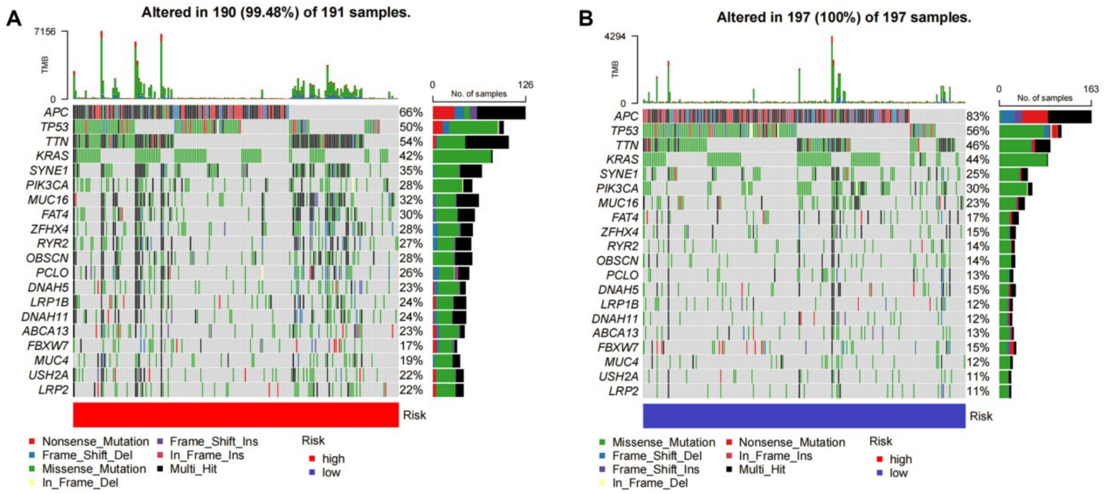

Supplementary Figure S4: Somatic mutations in the two risk groups of patients with COAD were compared. The five most mutated genes in the high- and low-risk groups were *APC*, *TP53*, *TTN*, *KRAS*, and *SYNE1*.
